# College Students' Learning Performance, Teaching Skills, and Teaching Innovation in Intercultural Communication Class: Evidence Based on Experiential Learning Theory

**DOI:** 10.3389/fpsyg.2022.953501

**Published:** 2022-07-28

**Authors:** Xueli Zhang, Xiaoyan Cheng

**Affiliations:** ^1^Foreign Languages Department, Taiyuan Normal University, Jinzhong, China; ^2^Management Department, Taiyuan Normal University, Jinzhong, China

**Keywords:** learning performance, teaching innovation, abstract conceptualization, teaching skills, intercultural communication class

## Abstract

In China, the improvement of the learner performance is critical a challenge for the teaching staff and the management in intercultural communication class. Indeed, the administration of the Chinese schools is failed to provide effective learning to the students with innovative methods. The objective of this study was to identify the role of college students' learning performance, teaching skills, and teaching innovation in intercultural communication class. This study is based on the quantitative data collected on a five-point Likert scale from the target respondents who were the students of different colleges and 700 questionnaires distributed for it. The study concludes that there is a significant relationship among abstract conceptualization, active participation, and reflective observation in students' learning performance. Furthermore, this study highlights that the mediating role of teaching innovation is critical for students' learning performance. This study contributes to the literature with a significant theoretical framework. Finally, this study provides significant theoretical implications and practical implications which are key game-changers for improving the performance of the students in the intercultural communication class.

## Introduction

In the current era, effective communication is considered one of the reliable factors for developing intercultural communication during the classes of students in the educational institute (Reinholz et al., 2022). However, it is also noted that traditional methods of teaching are not critical in modern studies, because the students are not getting the appropriate ability from the course material to improve their performance (Khoshnoodi Far et al., 2019). Moreover, in developing countries, innovative methods of teaching are considered reliable sources for providing the best opportunity to the students for effective learning (Sormunen et al., 2020). Importantly, it is noticed that the conceptual understanding of the students is not polished by the teachers, and this is one of the hurdles in the performance of the students (Aguillon et al., 2020). Similarly, the teaching institutes are designed to develop the mental ability of the student to provide them with effective and understandable information and build their capacity for effectively getting the broader goals (Okada, 2021). Furthermore, in backward countries, there are fewer opportunities provided to the students regarding the abstract conceptualization and active experimentation. In this way, it has become a hurdle in the performance of the students of China because they are still facing issues in the remote areas as the students are less treated by the stakeholders for developing their critical ability (Muhyiddin and Zharfa, 2018).

Reflective observation is important to consider in the modern methods of teaching because the students are expected to learn in the best for their better development (Muhyiddin and Zharfa, 2018; Reinholz et al., 2022). Indeed, in the traditional learning system, the role of modern and developed parameters of learning was neglected because there were limited resources and opportunities (Freguia, 2017). Similarly, according to Reinholz et al. (2022) in the modern learning methods, the concept of active experimentation has emerged that is helping the students with practical implications of the learning material for the achievement necessary for sustainability in learning. It is noted that the students with practical application of the ideas are more relaxed and understanding of the environment. Also, abstract conceptualization helps the students to develop the concepts and conclusions for more reliable and experience-based information (Freguia, 2017). It is the critical understanding development in the innovative teaching methods that were not addressed in the traditional time. Moreover, the innovative teaching methods are improving the quality of education for the students because the focus of these methods is students-oriented (Fullana and Pallisera, 2019). Importantly, according to the study of Reinholz et al. (2022) student learning performance is critical to consider because it provides the opportunity for the students to develop the abilities for better grooming in the modern world. However, very less focus was considered on the effective performance of the students in the previous traditional methods of teaching.

The objective of this study was to identify the role of college students' learning performance, teaching skills, and teaching innovation in intercultural communication class. It is noted that very few earlier studies have discussed the relationship between these variables that are presented in the framework of the study of Bush et al. (2022). In this way, this study aims to provide a clear picture of the students' learning performance and the factors that are influencing in it the modern world. Moreover, this study also emphasizes determining the important role of teaching innovation and abstract conceptualization to determine the influence of these factors on the critical learning performance of the students as discussed in the study of Nguyen et al. (2022). Furthermore, this study is designed to provide a significant contribution to teaching innovation as a mediator between the relationships among active experimentation, abstract conceptualization, reflective observations, and students' learning performance. Also, this study is to address the theoretical gap in the literature and practice gap in actual practice for the improvement in the performance of the students in learning as critical learning. Therefore, this study aimed to define the relationship between different variables and design these all in a productive way to facilitate the students in learning performance.

The study has significant importance because it provides both theoretical and practical implications regarding the role of college students' learning performance, teaching skills, and teaching innovation in intercultural communication class as highlighted by the study of Bush et al. (2022). During the literature review, it was identified that there is a significant gap in the literature because no earlier study has been conducted to determine the mediating role of teaching innovation in the relationship among active experimentation, abstract conceptualization, reflective observations, and students' learning performance. Similarly, different studies highlight the problems in practices related to the role of abstract conceptualization and reflective observations in students' learning performance in intercultural communication class (Mahlambi, 2021). In this way, this study is designed to provide sustainable theoretical and practical implications for the achievement of the students' learning performance with the relationship between different variables such as abstract conceptualization, reflective observations, and active experimentation. Importantly, this study provides the detailed results and the relationship between different variables. Additionally, this study would provide significant future directions for the coming studies to provide more reliable factors for students' learning performance.

## Literature Review

**Figure 1:**
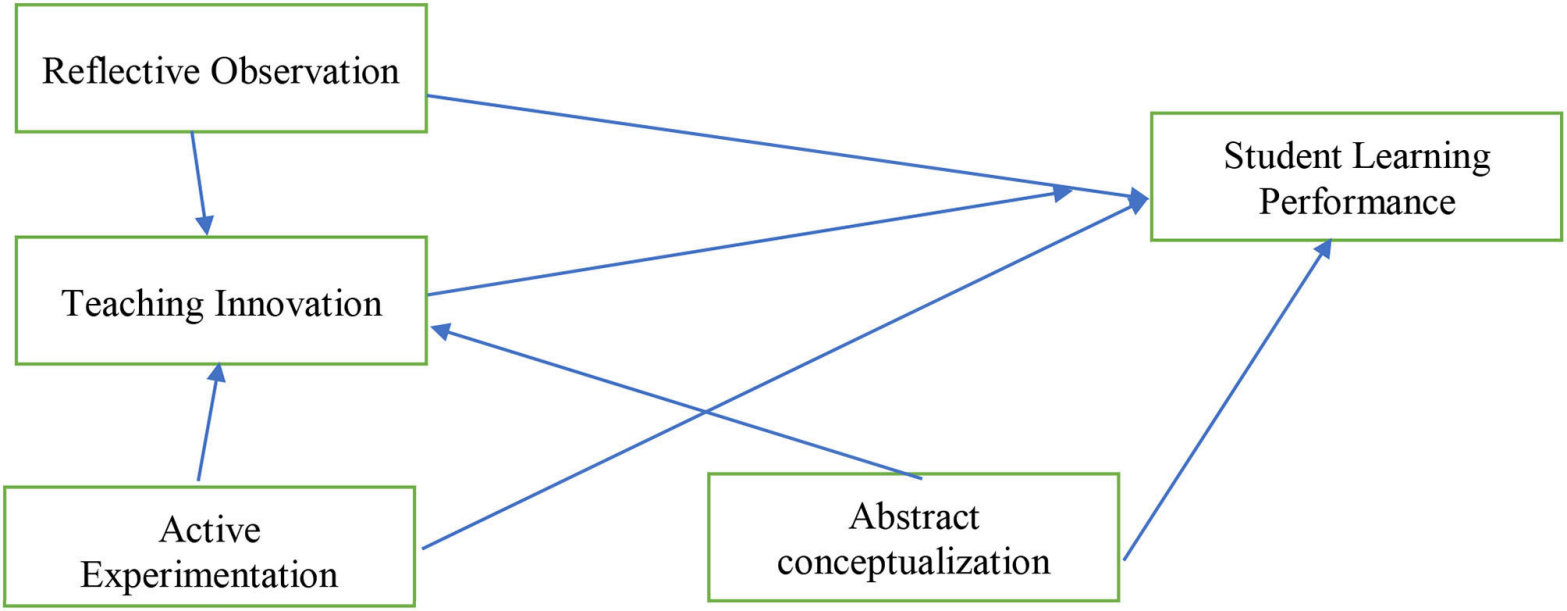
Theoretical framework. RO, reflective observation; TI, teaching innovation; AE, active experimentation; AC, abstract conceptualization; SLP, students' learning performance.

The theoretical framework (refer to Figure 1) for this study was supported by the theory of experiential learning presented by John Dewey. According to Dewey, knowledge is socially constructed information based on the experience we have in society (Bontchev et al., 2018). It is noted that this theory provides the real sociocultural system as an important factor for learning. Indeed, this theory was presented in the best regard to provide a detailed insight into experimental learning (Morris, 2020). Furthermore, this theory emphasizes that the knowledge should be organized with real-life experience because the context of the information is important in learning (Kuk and Holst, 2018). Based on this theory, the framework of the study provides multiple other factors such as reflective observation, abstract conceptualization, and active experimentation as the factors to affect the student's learning performance as this theory deals with learning performance. In this way, it would be interesting to retire that active experimentation is critical in learning as presented in the theory that the information is always obtained from a particular context. Therefore, this theory helps for the better development of the learning performance of the students with the actual contribution of the environment to the behavior of the student.

### Relationship of Reflective Observation, Teaching Innovation, and Students' Learning Performance

**Figure 2:**
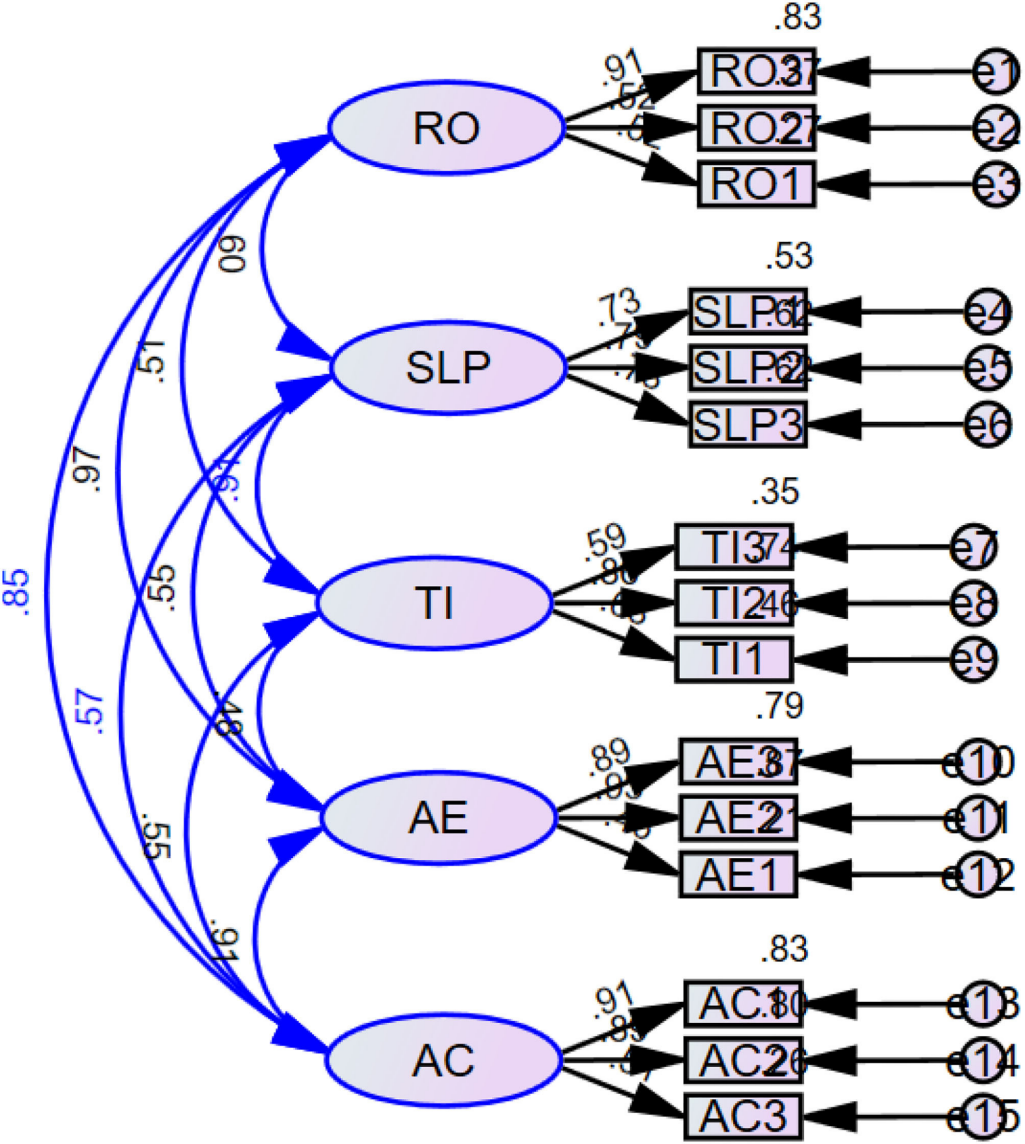
Measurement model.

Teaching is one of the critical methods that are important for the students to provide all the information related to the course material and educational performance (Widanta et al., 2019). In past, teaching was not strategically analyzed by different teachers in different institutes because it was believed that students must be provided with the cost material in any way (Smith et al., 2022). However, this concept was changed the time due to the emergence of different global institutes that contributed a lot to the development of teaching for the productivity and constructive learning of the student (Huang et al., 2022). In this regard, the different institutes have taken different initiatives to provide new and effective teaching methods to the students, because it is believed that students have a dynamic profile and they belong to different kinds of values and mindsets (Laletina et al., 2022). In this way, if the students are provided with different digital methods that are effective for them, then it would be great for the development of different strategies to improve their learning performance of the students (Huang et al., 2022). Moreover, for the teaching method improvement, the students must be provided with an opportunity with the critical learning standard to improve their performance of the student regarding their teaching material (Cilliers et al., 2022). In the advance and the developing countries such as Japan and China, the opportunities for innovative teaching are provided to the students because it is expected that with that kind of opportunity, the teachers are improving the standard of living if they are introducing the innovative method in teaching to facilitate the students to the advance level (Bush et al., 2022). Similarly, with this advanced kind of strategy, it would be expected that the teacher would play an important role in the critical learning of the students. Not only, in the advanced countries but in the modern time, in the backward countries, opportunity for critical learning is provided to the students by the teachers to improve their standard of learning because it is believed that if the students are provided with innovative teaching methods, then they would be able to think out of the box (Widanta et al., 2019; Asmus, 2021). All students have different mindsets and they have different skills and abilities to cope with different kinds of problems that are hurdles in the way of teaching (Hernandez-Olivan et al., 2022; Meng and Yang, 2022). Importantly, the role of teachers is critical in the development of students because the students are playing an important role in the development of the country, and when they are provided with an innovative method of teaching, then this opportunity would boost the learning ability of the students. Indeed, the teachers that are highly skilled and they are providing opportunities for critical learning to the student for their better observation and understanding, the teachers are providing the best for the student to improve their critical ability and understanding related to the course material (Nierenberg, 1998; Kosiba et al., 2019). It is also expected from the teachers that they are not only responsible to provide the opportunity related to the learning performance, but they must have to develop the students regarding their extra activities related to the study material that would be helpful for them in the long run (Hernandez-Olivan et al., 2022; Meng and Yang, 2022). The importance of critical learning is for the teacher to provide more opportunities in an innovative way to the students to ensure that they are learning according to the latest requirement of the market (Mulongo, 2013). The traditional teaching methods were required in the previous time but these are outdated and have no importance in the modern time because now, the dynamics of the market have been changed, and it is the responsibility of the teachers to provide an innovative method of teaching to drill the force material and others in the students to improve their understanding. In America, teachers are applying these methods in the classroom for the students because it is believed that when the method would be applied effectively, the greatest outcome would provide a better opportunity for the students to learn the thing innovatively (Widanta et al., 2019; Yao, 2019). Therefore, not only the teachers are required to utilize the innovative method for the student, but also it is the responsibility of the student to develop their mindset and adopt all of the new and innovative methods of teaching with a great heart and get the learning material effectively to improve the performance and the standard of education (Hernandez-Olivan et al., 2022; Meng and Yang, 2022). Moreover, the teachers are highly motivated and they have always in a race to innovate the new and emerging methods of teaching for the students, and these teachers are providing critical opportunities to the students for their bad learning (Laletina et al., 2022). For the development of the student's performance and the students' learning material, the responsibility of the teacher is to understand the mental state of the student and development of a strategy for the effective application to influence the mental state of the students for providing them the course material (Widanta et al., 2019; Sormunen et al., 2020). It is important to understand that for the development of the student's performance and the students' learning material, the responsibility of the teacher is to understand the mental state of the student and development of a strategy for the effective application to influence the mental state of the students for providing them the course material in an effective way (Widanta et al., 2019; Sormunen et al., 2020). The more effective course material would be provided to the students, and as a result, the more productivity would be developed in the student if the teachers are using non-traditional and new methods of teaching (Hernandez-Olivan et al., 2022; Meng and Yang, 2022). We have developed hypothesis based on Figure 2.

*H1. There is a relationship between reflective observation and teaching innovation*.
*H2. There is a relationship between reflective observation and student's learning performance*.
*H3: There is a relationship between teaching innovation and student's learning performance*.

### Relationship Between Active Experimentation, Teaching Innovation, and Student's Learning Performance

It is critical to understand that in modern times, the development of technology and other factors help to change the dynamics of teaching, and teaching has become a more focused and innovative behavior for the students and the teachers as well (Nyqvist et al., 2022). It is due to the reason that in past, teaching was considered a normal way of providing the course material and class activities to the students for their understanding. However, according to the study of Widanta et al. (2019) with the development of technology and development, it is need of the hour that the students must be provided with the effective and efficient skills that are critical for their better learning to compete with the globalized world and the students of the other institution. In this way, it is the responsibility of the stakeholder to innovate the new way of teaching to provide the course-related material to the student for a better understanding and their battle communication with learning skills in the classroom (Widanta et al., 2019; Smith et al., 2022). Indeed, the responsibility of the management is to provide an effective way of learning to the students but the most important factor in that teaching method because until and unless a teacher is not providing an innovative method of teaching to the students it would be completely difficult for the student to adopt the new changes with proper motivation (Saddhono, 2018; Peng et al., 2022). In this regard, the teachers are expected to provide a more practical approach to the teaching material and the course material related to the class activity to the students to ensure that the students are getting effective because it is a matter of their learning and their better understanding that would be helpful for them to competing with the students of the other institutions (Widanta et al., 2019; Asmus, 2021; Bush et al., 2022). It is a critical success factor if the students are provided with the opportunity of learning innovatively and they are assigned the task of class activities to communicate with each other and solve the problem with critical understanding by mutual sharing of ideas with the other students to provide effective results. However, this responsibility is not limited to the critical learning of the students, but it would affect the students to a greater extent and provide them away for better understanding and better learning to innovate the situation in which critical learning would be important for the students and the teachers as well, providing the opportunity of political learning, and it would be more important for them to deal with the critical education to the student for the better learning (Gutstein, 2007; Xia et al., 2022). However, in modern times, it is also expected that the students must not learn the course material with an objective, but they are provided with the opportunity for the implication of that course material for their better understanding and getting the right information for the productivity (Widanta et al., 2019; Yao, 2019). In critical learning ability, the focus is on the experimentation of the learning course material because until and unless the implication is not a procedure, it would be used to get the education and learn the course material for the students (Taylor et al., 2019). This opportunity is provided to the student by the government of Canada that the students are experimenting with their learning course material with the help of teachers because these teachers in their critical learning and development of the understanding to the advanced level. No doubt, if such kind of critical learning of authorities is provided to the students and they are provided with the opportunity of development and implication of their course material with the help of teachers, it would result in battle productivity and improve the performance of the students (Laletina et al., 2022; Nazari and Alizadeh Oghyanous, 2022). Therefore, it is considered that the students must be provided with the critical learning ability and they must have all kinds of motivation and information related to the course material to implement this understanding in a productive way to get a better outcome. The educational system in Korea is designed in a way that is providing the opportunity for innovative teaching methods to the students to introduce the new and alternative ways of teaching for the learning of the course material and adopt the related material with efficient understanding (Maretto et al., 2020; Laletina et al., 2022). Furthermore, this course material is not for just learning, but it is also expected that the students would get a better understanding and they would learn the course material by heart when they are provided with different types of critical learning skills to improve their standard of understanding and better experiment (Laletina et al., 2022). Similarly, in China, it is believed that until and unless the students are not participating in active experimentation with the learning course material in result, and it would be difficult for the student to develop the critical learning ability to the advanced level (Hernandez-Olivan et al., 2022; Meng and Yang, 2022). It is noted that the performance of the students related to the learning material is directly dependent on the environment that is provided to the student for learning. In this regard, it is important to consider that if the students are provided with an effective learning environment in result, and the performance of the student would be increased to an advanced level (Chen et al., 2022). However, on the other hand, if the students are not provided with a suitable environment for learning in result, it would be difficult for them to develop the strategies for effective learning and this would damage their critical learning performance of the students (Ayuningtyas et al., 2022). Therefore, students must be considered as one of the effective factors responsible for the society because if the educational system improves the understanding of the student and provides the opportunity for active participation, it leads the educational system to the advanced level. In India and Nepal, the educational system is not the expected advanced requirement of the metal educational system that is providing the opportunity to the student for better learning and improving their critical learning ability for their better performance (Onan, 2020; Laletina et al., 2022). As a result, the students of these countries are not comparable as efficient according to the students of modern countries, and the students are left protective and less understandable because they are not provided with an opportunity for active participation. Indeed, it is the responsibility of the ministry of education in India to ensure that the students are provided with the alternative way of teaching methods to improve their battle understanding for learning the course material that would help their critical learning ability by actively participating and improving the standard of learning performance (Saldana Corral, 2019; Chotimah et al., 2021).

*H4. There is a relationship between active experimentation and teaching innovation*.
*H5. There is a relationship between active experimentation and students' learning performance*.

### Relationship of Abstract Conceptualization, Teaching Innovation, and Students' Learning Performance

In modern times, the teaching method is changed because the students are new and they are requiring the alternative and best teaching method for better understanding. It is critical to understand that with every generation, the teaching method has to be changed because the people are of a different mindset, and they have a different set of values regarding the learning performance and critical learning ability (Widanta et al., 2019; Laletina et al., 2022; Smith et al., 2022). However, according to Laletina et al. (2022), it is the responsibility of the teachers to provide the best alternative method of teaching to the students if they are not getting the related course material with the help of the traditional method of teaching. Therefore, the most important problem that occurs in this way is that it is difficult to classify the students into different categories and select different teaching methods for each category because the human critical learning ability is not easy to detect and understandable for any category by the teachers. In this regard, the teachers in Australia and Canada are working to categorize the students into different categories and developed the critical learning ability for the students of each category to ensure that they are provided with all-related information according to their mental level (Hartono et al., 2022; Laletina et al., 2022). However, this is not limited to the developed countries, and in the developing country, the teachers are working to improve the critical learning ability of the students with a more understandable and affordable way of teaching (Mårtensson, 2020). In this way, it is the responsibility of the stakeholders to understand to what extent the active conceptualization method must be integrated with the new innovative teaching method for the students to improve their critical learning ability of the students (Widanta et al., 2019; Laletina et al., 2022). In the past, it was believed that critical learning ability improves the student's critical mindset but in modern time, it is determined that critical learning also influences the performance of the students who are learning (Stokes et al., 2020). Moreover, it is also understood that the students who are provided with an opportunity for effective learning and are guided by their teacher to be active in this conceptualization are more innovative and they have adaptive behavior to digest the innovative teaching method. On the one hand, the responsibility of the stakeholders is to provide the appropriate resources to the teachers for the implementation of effective teaching methods for the students to improve their learning ability to the advanced level (Widanta et al., 2019; Putri et al., 2020). However, on the other hand, it is the responsibility of the teachers to invite the new ideas and implement them in the teaching method for the students because different students have different learning abilities and they are from different backgrounds. The critical responsibility of the students is to respond to the guidelines of the teachers and determine their weaknesses to convert the weakness into a strength to motivate themselves for improving their critical learning ability that would affect their performance (Widanta et al., 2019; Sulistyarini et al., 2022). Importantly, the students are not provided with an effective learning method and are less adaptive for the improvement of their critical skill; in this regard, the new and innovative teaching method is useless for them to be introduced because they are not motivated to integrate with this all methods of innovative teaching. In the traditional concept of the study, the students were provided with the opportunity to learn the books and get the learning material with the motivation of the teachers (Saddhono, 2018; Widanta et al., 2019). However, in China and Japan, the teachers are not only motivating the students for the new innovative method, but also they are influencing the critical and adaptive behavior of the students to accept all the changes in the critical learning ability that are required for improvement in the standard of teaching. Similarly, in the advance and developed countries, the focus of the teachers is to provide the course material with the methodology of active conceptualization because it is understood that if the students are provided this opportunity, then it would be reasonable to learn the course material by getting more information and providing more relevant understanding (Lee et al., 2013; Widanta et al., 2019; Laletina et al., 2022). The idea of abstract conceptualization is developed in the teaching method because with the help of this idea, the opportunity is provided to the students to get all the related materials for the better development because until and unless they are not provided with the opportunity to learn critically and developed the concepts and conclusion with the event, it would be useless for them to study in the modern time. Importantly, it is the responsibility of the stakeholders to integrate the traditional learning pattern with the modern learning opportunities to provide more effective and efficient critical learning ability to the students to improve their understanding of abstract conceptualization (Gan et al., 2020; Hu et al., 2022; Laletina et al., 2022). It is important to understand that abstract conceptualization provides the opportunity for the students to integrate the learning material with a better understanding to apply the theories and concepts to develop a conclusion for a better understanding. Moreover, it is important to understand that the performance of the learner is dependent on different factors that are important to consider and the most important factor is the teaching method (Widanta et al., 2019; Laletina et al., 2022). The innovative teaching methods are providing different alternative opportunities to the students for the better development of the concept and abstract conceptualization that improves the standard of critical learning ability of the students for their better understanding of the ideal presented in the course material (Ghasemian Safaei and Farajzadegan, 2012). Furthermore, if the teachers are provided with the battle opportunity and they are having the ability to implement the innovative method, then the loyal and responsible teachers are adopting the innovative method for developing the more focused and the advanced concept in the students for their better learning. The abstract conceptualization concept is important for the students because it provides them with a way of learning that was not presented in the traditional time, and it is highlighted in the modern time that helps them to conclude the concepts with the help of perception and intuition (Mulongo, 2013; Saddhono, 2018). The responsibility of the student is to become an efficient learner and adopt all of the critical skills and study material in an effective way for the better development and understanding that would lead them to the advanced level of success. Importantly, the students must focus on their critical learning ability for their learning, and they must have to improve their performance learning with the help of innovative ideas for better development of their critical skills with the help of supportive teachers.

*H6. There is a relationship between abstract conceptualization and teaching innovation*.
*H7. There is a relationship between abstract conceptualization and students' learning performance*.
*H8. There is a mediating role of teaching Innovation in the relationship between reflective observation and students' learning performance*.
*H9. There is a mediating role of teaching innovation in the relationship between active experimentation and students' learning performance*.
*H10. There is a mediating role of abstract conceptualization in the relationship between reflective observation and students' learning performance*.

## Methodology

### Prepare Questionnaire

In this study, the data were collected with the Likert scale questionnaire that was developed with the contribution of research experts. For survey design research, five-point Likert scale questionnaire is appropriate to consider because it is easy to understand by the respondents. Furthermore, with this type of questionnaire, data can be collected in a given time frame because this method is appropriate to collect data from the large number of populations. The questionnaire carried scale items for each variable that were to be tested by AMOS data analysis tool. First, the scale items for reflective observation were adapted from the study of Kember et al. (2000). Second, the scale items for teaching innovation were adapted from the study of Chou et al. (2019). Third, the scale items for active experimentations were adapted from the study of Allinson and Hayes (1990). Fourth, the scale items for abstract conceptualization were adapted from the study of Volkova and Rusalov (2016). Finally, the scale items for students' learning performance were adapted from the study of Hughes et al. (2012). Importantly, these scale items were adapted and reviewed by the experts before presenting the questionnaire to the target respondents. In this regard, after getting the positive response, the questionnaires were distributed to the respondents.

### Data Collection Process

In this study, the data were collected on Likert scale questionnaire because the desire was to collect the quantitative data as the other studies similar to it have used quantitative data for the measurement of the scale items for each variable. Furthermore, it was identified that the survey method of data collection is appropriate when the data are to be collected from a large population. The sample size for this study was 320 as the earlier studies have used the average sample size of 310 individuals. The target population of this study was the students of different colleges and the teachers in Wuhan city, and the expected response rate was 40%. Therefore, 700 questionnaires were mailed to the target respondents with the paid return envelope to facilitate them. Importantly, the introduction of the study was provided to them along with the questionnaire because the intention was to provide them a brief about the study before responding to the questionnaire. Additionally, the email was provided to the respondents for any kind of questions related to the questionnaire. In this way, the respondents were provided with the answers of their queries on time. After 1 week, the questionnaires were collected back for final measurements. Unfortunately, 16 questionnaires out of 336 received questionnaires were incomplete; therefore, it got rejected. However, the considered 320 questionnaires were appropriately taken for the collection of data. Ethically, the respondents were thankful for their interesting and worthy contribution. This procedure of data collection was considered as the earlier studies conducted in the same area deals with the same sampling technique.

## Findings

### Measurement Model

This section provides the results of confirmatory factor analysis (refer to Table 1). It is critical to understand that the confirmatory factor analysis is conducted to measure the factor loadings and the scale items for each variable of the study. According to the values, all of the scale items except one have factor loadings > 0.50, which is recommended by Wong (2013), for the future and advance studies. Moreover, in the same way, the scale items for each construct are presented in this section. In this regard, three scale items were taken for reflective observation, three scale items for students' learning performance, three scale items for teaching innovation, three scale items for active experimentation, and three scale items for abstract conceptualization to test the relationship between different hypotheses.

**Table 1:** Results of confirmatory factor analysis.

**Constructs**	**Scale items**	**Standardized factors loadings**
Reflective observation	I sometimes question the way others do something and try to think of a better way.	0.911
	I like to think over what I have been doing and consider alternative ways of doing it.	0.518
	I often reflect on my actions to see whether I could have improved on what I did	0.521
Students' learning performance	My teacher conducts him/herself professionally	0.728
	My teacher makes positive comments about the student's abilities to learn	0.79
	My teacher is patient with students when directing them to learn appropriate behaviors	0.785
Teaching innovation	I can become familiar with the contents of ICT different units.	0.594
	I can prepare teaching ICT materials and tools for different units	0.858
	I can change ICT instructional activities to maintain students	0.682
Active experimentation	I thrive on the challenge of something new and different	0.888
	I am careful not to jump to conclusions too quickly	0.934
	I believe that rational, logical thinking should win the day	0.463 (deleted)
Abstract conceptualization	I suggest many versions of solving problems in complex tasks	0.91
	I always prefer alternatives ways of problem solving	0.895
	I understand problem solving is easier by thinking out of box	0.511

Similarly, the reliability and validity test were conducted to test the reliability of the scale items for each variable according to the context of the study. According to the results presented in Table 2, there is a clear reliability and validity because the value of CR was >0.70, which is recommended by the study of Wong (2013) for each variable, and the value of AVE was >0.60 for each variable. Therefore, the results reveal the reliability and validity between the scale items of the study.

**Table 2:** Reliability, validity, statics, and correlations.

	**CR**	**AVE**	**MSV**	**MaxR(H)**	**RO**	**SLP**	**TI**	**AE**	**AC**
RO	0.744	0.656	0.942	0.849	**0.676**				
SLP	0.812	0.690	0.821	0.814	0.597***	**0.768**			
TI	0.759	0.618	0.821	0.808	0.513***	0.906***	**0.720**		
AE	0.823	0.625	0.942	0.916	0.970***	0.550***	0.485***	**0.791**	
AC	0.829	0.630	0.832	0.902	0.846***	0.570***	0.552***	0.912***	**0.794**

*RO, reflective observation; TI, teaching innovation; AE, active experimentation; AC, abstract conceptualization; SLP, students' learning performance*.

*Bold values indicate the results for corresponding statistics for whole variable not the items*.

^***^means significant at 1%.

Moreover, the measurement model fit was analyzed by evaluating the root mean square of approximation, absolute fit measures, standardized root mean square residual, comparative fit index, normed fit index, and adjusted goodness of fit. In this regard, the recommended threshold was achieved for evaluating the model, and the values are presented in Table 3.

**Table 3:** CFA model.

**Measure**	**Recommended threshold**	**Abbr**.	**Scores**
Chi-square/df (CMIN/DF)	<3.0	2/df	2.111
Comparative fit index	>0.90	CFI	0.8
The normed fit index	>0.90	NFI	0.87
Goodness of fit	>0.90	GFI	0.87
Adjusted goodness of fit	>0.80	AGFI	0.79
Root mean square residual	<0.08	RMR	0.09
Standardized root mean square residual	<0.08	SRMR	0.08
Root mean-square error of approximation	<0.08	RMSEA	0.08

### Discriminant Validity—HTMT

In this section, the discriminant validity of the scale items was tested (refer to Table 4). The discriminant validity is used to test the distinction between the variables and the scale items used in the theoretical framework of the study. According to the results, all the values of HTMT discrimination validity method were <0.90, which is recommended for the modern and advance studies. Moreover, the results reveal that there is a clear discriminant validity between the scale items and data collected for this study.

**Table 4:** Discriminant validity (HTMT).

	**RO**	**SLP**	**TI**	**AE**	**AC**
RO					
SLP	0.668				
TI	0.684	0.704			
AE	0.764	0.680	0.777		
AC	0.882	0.641	0.679	0.765	

*RO, reflective observation; TI, teaching innovation; AE, active experimentation; AC, abstract conceptualization; SLP, students' learning performance*.

### Structural Model

In this section, the results of the hypotheses are presented (refer to Table 5). H1 was tested to check its significance, and according to the results, reflective observation has a significant effect on teaching innovation (β = 0.271, *t* = 3.912, *p* = 0.000), and H1 is accepted. H2 was tested to check its significance, and according to the results, reflective observation has a significant effect on students' learning performance (β = 0.233, *t* = 3.618, *p* = 0.000), and H2 is accepted. H3 was tested to check its significance, and according to the results, teaching innovation has a significant effect on students' learning performance (β = 0.211, *t* = 3.712, *p* = 0.000), and H3 is accepted. H4 was tested to check its significance, and according to the results, active experimentation has a significant effect on teaching innovation (β = 0.271, *t* = 3.699, *p* = 0.000), and H4 is accepted. H5 was tested to check its significance, and according to the results, active experimentation has a significant effect on students' learning performance (β = 0.281, *t* = 2.912, *p* = 0.000), and H5 is accepted. H6 was tested to check its significance, and according to the results, abstract conceptualization has a significant effect on teaching innovation (β = 0.271, *t* = 5.212, *p* = 0.000), and H6 is accepted. H7 was tested to check its significance, and according to the results, abstract conceptualization has a significant effect on students' learning performance (β = 0.471, *t* = 4.324, *p* = 0.000), and H7 is accepted. According to the results of H8, teaching innovation mediates the relationship between reflective observation and students' learning performance (β = 0.112, *t* = 4.123, *p* = 0.000), and, H8 is accepted. According to the results of H9, teaching innovation mediates the relationship between active experimentation and students' learning performance (β = 0.087, *t* = 3.977, *p* = 0.000), and, H9 is accepted. According to the results of H10, teaching innovation mediates the relationship between abstract conceptualization and students' learning performance (β = 0.098, *t* = 3.812, *p* = 0.000), and, H10 is accepted.

**Table 5:** Standardized path coefficient.

**Hypotheses**	**Relationship**	**Beta**	***t*-values**	***p*-values**	**Decision**
H1	Direct	0.271	3.912	0.000	Accepted
H2	Direct	0.233	3.618	0.000	Accepted
H3	Direct	0.211	3.712	0.000	Accepted
H4	Direct	0.271	3.699	0.000	Accepted
H5	Direct	0.362	4.822	0.000	Accepted
H6	Direct	0.271	5.212	0.000	Accepted
H7	Direct	0.471	4.324	0.000	Accepted
H8	Mediation	0.112	4.123	0.000	Accepted
H9	Mediation	0.087	3.977	0.000	Accepted
H10	Mediation	0.098	3.812	0.000	Accepted

## Discussion

The results of H1 and H2 reveal that there is a significant relationship among reflective observation, teaching innovation, and students' learning performance. The performance of the students in the class activities is dependent on the contribution of the teacher and the behavior of the student (McGettigan and McKendree, 2015; MHum et al., 2016). The students who are highly motivated and have a deep observation regarding the understanding of course material are more motivated to learn as discussed in the study of Widanta et al. (2019). It is important to consider that the responsibility of the teachers is to contribute to the progress of the students by adopting different alternative methods of teaching and capacity building the students as discussed in the study of McGettigan and McKendree (2015) and Fabito et al. (2018). In this regard, if the management of college institutions is not highly innovative regarding the course material and the activities of the students in result, the performance of the students would be declined (Dong, 2020). Therefore, the teachers must innovate new methods of teaching according to the mental ability of the students to provide them with class material with effective communication that would help them to develop better strategies for learning as discussed in the study of Valentina et al. (2022). It is critical to determine that if the strategies are not supportive to the students in result, their learning performance of the students would be declined (Valentina et al., 2022). The results of H3 reveal that there is a significant relationship between teaching innovation and students' learning performance. It is important to understand that the students who are working hard for improving their performance if they ported by the innovative teachers, then it would be great for them to leave the critical things in an effective way for the development of their abilities according to the modern standard of education (McGettigan and McKendree, 2015; Brubaker and Beverly, 2020; Yan, 2021). In past, there was less focus on the innovation of teachers for the methodology of teaching, and it was considered an obstacle to the learning performance of the students as highlighted in the study of He and Chiang (2016). Therefore, the responsibility of the college administration is to motivate the teachers with innovative teaching methods to contribute to the profile of the student (Chang and Suttikun, 2017).

The result of H4 and H5 reveals that there is a significant relationship among active experimentation, teaching innovation, and students' learning performance. It is great to focus that the students are provided with the opportunity of relating their theoretical knowledge with practical active experimentation, and as a result, they develop their critical learning ability to an advanced level (Muhyiddin and Zharfa, 2018; Waheed et al., 2020). In Tokyo, the students are motivated to perform active experimentation related to the study material to develop their critical thinking ability and emphasize the importance of a practical approach to success (McGettigan and McKendree, 2015; Chang and Suttikun, 2017). On the other hand, the countries are badly failed to provide reliable and workable active experimentation opportunities for the students, and the college institutions of these countries are not capable of coping with the modern problem as discussed in the study of Valentina et al. (2022). The responsibility of the college administration is to provide practical opportunities for the students for active experimentation with the help of innovative teaching methods to build their capacity for learning and improve their learning performance (McGettigan and McKendree, 2015; Kosiba et al., 2019; Dong, 2020). In this way, this opportunity would be beneficial for the students as well as for the stakeholders who are devoted to enhancing the critical abilities of the students with the practical approach. The results of H6 and H7 reveal that there is a significant relationship among abstract conceptualization, teaching innovation, and students' learning performance. In the country, where the students are trained with the help of different types of workshops and sessions to improve their performance of learning, the students are performing well and are more innovative than the rest of the students as discussed in the study of Valentina et al. (2022). However, the critical responsibility of the administration is to integrate the course material for the students with the innovative teaching method to ensure that they are provided with all the relative information to get better observation (McGettigan and McKendree, 2015; Sîrbu et al., 2015; Kosiba et al., 2019). If the students are provided with opportunities related to the capacity-building classes, then as a result, the abstract conceptualization ability of the students would be developed and it would be more learning-oriented (McGettigan and McKendree, 2015). It is critical to understand that the students are having different qualities, but then, these qualities are polished with the help of effective management, which helps the student to be more conceptual and intercultural in the learning activities. For the capacity building and intercultural communication classes, the responsibility of the administration is to train the student for abstract consideration to conclude based on the experience and events (McGettigan and McKendree, 2015; Chang and Suttikun, 2017; Dong, 2020). In this way, the students would be more focused to improve their performance compared with the students of other institutions.

The results of H8 demonstrate the significant mediating role of teaching innovation between the relationship of reflective observation and students' learning performance. It is critical to determine that the responsibility of the teachers is to provide alternative ways in which the performance of the students can be improved. In China, the students are treated with innovative ways of teaching to be facilitated by the guidelines of the teacher, and it helps them to improve their performance (Angela, 2014; MHum et al., 2016; Kazemitabar and Garcia, 2021). It must be considered that the teachers are highly responsible to motivate the student by adopting different strategies to make them capable of learning and improving their performance (McGettigan and McKendree, 2015). Indeed, this strategy of capacity building and reflective of the students is quite helpful for the college students. The results of H9 demonstrate that there is a significant mediating role of teaching innovation between the relationship of active experimentation and students' learning performance. It is observed that the students who are provided with the opportunity of learning critically are more relaxed in learning performance as discussed in the study of Ghasemian Safaei and Farajzadegan (2012). On the other hand, the opportunity is important for the students because active experimentation makes them capable of learning in a controlled environment where the actual implications of learning help to enable the capacities for better grooming (Murray and Lang, 1997; Widanta et al., 2019; Hu et al., 2022; Reinholz et al., 2022). Therefore, the teachers need to improve the learning performance by adopting new environment ideas for enhancing the learning performance of the students (Reinholz et al., 2022). The results of H10 reveal that there is a significant mediation of teaching innovation between the relationship between abstract conceptualization and students' learning performance. The students of the modern institutions are more dependent on the innovative teaching approach because they believe that with new world learning methods, their performance would be increased (Brubaker and Beverly, 2020). However, if the teachers help the students for developing abstract conceptualization, it would be great for the students to learn new things in an alternative way (Niedzwiedz et al., 2021; Laletina et al., 2022; Nguyen et al., 2022). Therefore, educational institutions need to improve the quality of education and training in the learning performance of the students.

## Conclusion

The results of the study provide a significant conclusion that is important for a better understanding of the relationship among teaching innovation reflective observation, abstract conceptualization, active experimentation, and students' learning performance. It is critical to understand that this study demonstrates that there is an important mediating role of teaching innovation between the relationship of refractive observation and students' learning performance. The study highlights that if the reflective observation skills required to be provided to the students with the innovative teaching methods, then it would be more reliable for the students to contribute to the performance of the institution and the individual performance of the student themselves. Indeed, according to the study, the responsibility of the teachers is to provide every aspect for dealing with such kinds of critical issues to improve the performance of the learners with the help of innovative teaching methods that are dressing is a category of the student. Similarly, the results of the study demonstrate that there is a critical role of teaching innovation in the relationship between abstract conceptualization and students' learning performance. In this regard, it is observed that if the students are provided with effective teaching methods with innovative ways of learning their performance of the students would be increased, it is obvious that the students are learning in different colleges if they are provided with a teaching innovation approach, then the approach of the student related to the concepts would be increased for their better performance. It is critical to determine that the students need to address the issues effectively if they are provided with the more practical approach of abstract conceptualization in their learning. Finally, the study highlights that there is a critical role of teaching innovation in the relationship between active experimentation and students' learning performance. According to the results of the study, it is obtained that with the help of an innovative teaching method, it would be a more practical approach for the students if they are provided with the active experimentation approach to practice the learning material in actual life. Indeed, it is the right opportunity provided to the students when they are learning and providing the best output to contribute to the overall socioeconomic performance of the country. Therefore, it is expected that with the implications of the study, the stakeholders would consider the relationships between different variables to effectively improve the intercultural capabilities of the students in class.

## Implications

### Theoretical Implications

The conclusion of the study provides significant theoretical implications for the improvement in the intercultural communication class regarding the performance of the learner. It is important to understand that if the opportunity is provided to the students for their better improvement and productivity in-class activities with abstract conceptualization, then it would be more effective for the students to improve their performance with self-motivation. In this way, it is the responsibility of the stakeholder who is providing the guidelines and policy for the educational material and the methods of teaching because these guidelines would help the teachers to try a different way of teaching in improving the performance of the learners. It is a fact that if the teachers are working on the reflective observation with the help of innovative teaching methods, then a more focused education can be provided to the students for their better development. Similarly, no earlier study has discussed the mediating role of teaching innovation in the relationship between reflective observation abstract conceptualization and active participation to improve the student's learning performance. Therefore, this study highlights that this gap in the theory must be addressed with the guidelines related to the educational material which would be supported by the conclusion of the study to improve the performance of the learners. Besides, it is also important to consider that the educational institute in Tokyo is working to provide abstract conceptualization to the students for the performance of the learners because it is believed that the students need to improve their learning with their self-concept and draw the conclusion from the basis of different events. In this way, a more focused and more student-oriented teaching policy would help to improve the performance of the students to the advanced level by boosting their knowledge and improving their performance to make the standard of international education.

### Practical Implications

The results of the study also provide significant practical implications that are important to consider to improve the performance of the learners. It is critical to understand that in the backward countries, the educational institutes are badly failed to provide critical education to the students for their better self-concept building and integration with the markets of the modern time. However, the study provides significant implications that would address the problems if the stakeholders take these all implications for proper application in the educational institute, particularly in the college. This study demonstrates that the responsibility of the college management is to design the curriculum in an effective way that would be ready to absorb the hurdles on the way to success and provide new opportunities to the students. No doubt, in the traditional education system, the concept of reflective observation and active experimentation was not considered important. However, this study emphasizes that abstract conceptualization and active participation are important to integrate the abilities of the students with the actual performance that would help the student to grow productively. Moreover, the study highlights that the government and the non-government organizations should create awareness for the critical learning of the students and provide different alternative ways to integrate the innovative teaching method in colleges to provide standard education to the students to improve their performance of the learners. Similarly, it is also emphasized by the study that the management of colleges should monitor the activities of students and try to develop different alternative ways to communicate the best policy with the students for their intercultural development with the help of training and workshops. This study highlights that if the management of the colleges would be successful in conducting training and workshop for the student for intercultural development, then as a result, the performance of the learners would be increased. Therefore, it is the key responsibility of the management to ensure that the performance of the learners must be improved with the help of reflective observation technique abstract conceptualization technique and active experimentation technique with the radiating role of teaching innovation.

## Limitations

This study is purely based on the relationship among reflective observation, teaching innovation, abstract conceptualization, and active experimentation to improve the students' learning performance. Indeed, during the review of the literature, it was identified that multiple other factors can improve the performance of the students. In this way, future studies need to focus on the role of adaptive behavior in improving the performance of learners. Second, future studies need to identify the role of information communication technology in the learning performance of the students. Finally, the coming studies need to focus on the relationship between critical thinking and students' learning performance because it was not addressed by any past students in the context of intercultural communication class. Importantly, the role learning environment as a mediator must be considered in the relationship between reflective observation and student's learning performance.

## Data Availability Statement

The original contributions presented in the study are included in the article/supplementary material, further inquiries can be directed to the corresponding author.

## Ethics Statement

Ethical review and approval was not required for the study on human participants in accordance with the local legislation and institutional requirements. The patients/participants provided their written informed consent to participate in this study. The study was conducted in accordance with the Declaration of Helsinki.

## Author Contributions

Both authors listed have made a substantial, direct, and intellectual contribution to the work and approved it for publication.

## Funding

The work was supported by the Teaching Reform Project in Shanxi Provincial Education Department: Research and Practice on the Teaching Content and Teaching Design of College English Based on Curriculum Ideological and Political in Local Normal Universities (J20220932).

## Conflict of Interest

The authors declare that the research was conducted in the absence of any commercial or financial relationships that could be construed as a potential conflict of interest.

## Publisher's Note

All claims expressed in this article are solely those of the authors and do not necessarily represent those of their affiliated organizations, or those of the publisher, the editors and the reviewers. Any product that may be evaluated in this article, or claim that may be made by its manufacturer, is not guaranteed or endorsed by the publisher.
